# Analysis of the implementation of urban computing in smart cities: A framework for the transformation of Saudi cities

**DOI:** 10.1016/j.heliyon.2022.e11138

**Published:** 2022-10-18

**Authors:** Habib M. Alshuwaikhat, Yusuf A. Aina, Lolwah Binsaedan

**Affiliations:** a Department of City and Regional Planning and Interdisciplinary Research Center for Smart Mobility & Logistics, King Fahd University of Petroleum and Minerals, Dhahran, Saudi Arabia; b Department of Geomatics Engineering Technology, Yanbu Industrial College, Yanbu, Saudi Arabia; c Geoinformatic Unit, Geography Section, School of Humanities, Universiti Sains Malaysia, 11800 Penang, Malaysia; d Department of City and Regional Planning, King Fahd University of Petroleum and Minerals, Dhahran, Saudi Arabia

**Keywords:** Urban computing, Smart city, Urban informatics, Urban transformation, Urban sustainability, Saudi cities

## Abstract

Smart city development is gaining widespread acceptance as a means of mitigating urban development problems. However, the implementation of smart cities faces challenges, especially in developing countries. Urban computing is regarded as an enabler of innovation and smart city development. This study explores the adoption of urban computing to address the smart city and urban development problems of Saudi cities. Using a systematic review, this study highlights the trends and influential contributions of urban computing to smart city research. It identifies the urban computing framework and uses the framework to analyze the use of urban computing in Saudi cities to promote smart city development and sustainability. While Saudi Arabia has taken notable steps in urban computing, especially in providing services, further steps need to be taken to achieve the transformation into smart sustainable cities.

## 1 Introduction

Cities, globally, are facing unprecedented sustainability challenges as they become the major centers of environmental and socio-economic problems. Urban areas consume over 70% of the world’s resources, making them a major energy consumer of and contributor to greenhouse gas emissions (UN Habitat, 2019; Hong et al., 2020; Leavesley et al., 2022). Meanwhile, the population of the cities continues to rise. According to the United Nations (UN), 55% of the world’s population lives in cities and the proportion is expected to increase to 68% by 2050 (UN DESA, 2019). This fast urbanization is further compounding urban challenges, including traffic congestion, massive construction waste, and environmental pollution – air, noise, and water (Wang et al., 2014; Abubakar and Dano, 2020). These challenges require urgent steps to protect the urban environment and restrict the wasteful attitude towards natural resources. Global leaders and researchers have risen to the challenge by introducing sustainable development goals (SDG) and smart sustainable city initiatives.

Smart sustainable cities use varied technologies to attain a sustainable lifestyle and a constant, healthy quality of life. It is composed of information and communication technologies (ICT) that are primarily used to create, deploy, and promote sustainable development principles. This is regarded as a “new techno-urban phenomenon” (Bibri and Krogstie, 2017: 193).

Smart cities have six characteristics – smart economy, smart people, smart governance, smart transportation, smart environment, and smart living (Giffinger et al., 2007; Batty, 2013). All these are accomplished through smart technological solutions and ICT infrastructures, including the Internet of Things (IoT), big data, and cloud computing (Bibri, 2022; Kaginalkar et al., 2021; Thales, n.d.). However, it is important to remember that smart technologies are only a tool for supporting the drive toward sustainability in cities; they must not be the end goal.

A smart city is a critical initiative that all leading cities must implement, adapt, and operate. However, there are unique challenges to implementing smart cities in developing countries (Alqahtani et al., 2021; Tan and Taeihagh, 2020). It has been acknowledged that urban computing can facilitate the development of smart cities and improve their performance (Bibri, 2021). Saudi Arabia is one of the countries that have established smart city initiatives to address its urban challenges (Aina, 2017; Aina et al., 2019a). Smart city projects have been initiated in cities such as Riyadh, Yanbu industrial city, Makkah, Jeddah, Madinah, Al-Ahsa and Neom city (Doheim et al., 2019). The value of the Saudi smart cities market was estimated to be about $3500 million in 2019 (Gaul, 2021). Saudi Arabia has a rapidly growing urban population, which requires the cities to expand at a fast pace. The rapid population growth, combined with a lack of planning frameworks and weak city institutions unable to adequately control the growth, has resulted in sprawling and lopsided development (Aina et al., 2019b; Abubakar and Dano, 2020). Rapid spatial expansion of Saudi cities has led to several consequences, including traffic congestion, lack of transportation means, rapid consumption of natural resources, and lack of cultural awareness of these serious issues (Abubakar and Aina, 2018). These have environmental implications – high consumption of energy, production of greenhouse gases, constant air, noise, and water pollution – that need to be addressed to make Saudi cities sustainable.

There is a need to explore possible ways of mitigating the negative impacts of rapid urbanization in Saudi cities. This study aims to explore the use of urban computing to address the challenges of Saudi cities and improve the implementation of smart cities. The study aims to (1) map the trends and influential contributions of urban computing to smart city development, (2) identify the framework for urban computing, (3) use the framework to explore and analyze the adoption of urban computing in Saudi cities, and (4) identify the challenges of using urban computing to promote smart cities.

## 2 Methodology

**Figure 1:**
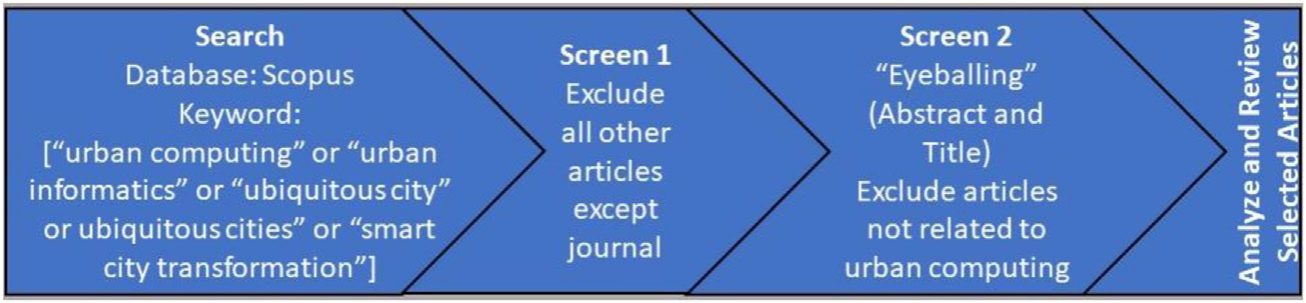
The flow diagram of searching and screening the articles in the Scopus database.

This study is based on literature review to identify the trends and influential contributions of urban computing to smart city research and analyze its use in Saudi Arabia for smart city development. The literature review was carried out in two parts. The first part was implemented through the Scopus database by using chosen search terms (urban computing or urban informatics or ubiquitous city or ubiquitous cities or smart city transformation) in February 2022. The search resulted in 1279 documents. Thereafter, conference proceedings, book series, books, and trade journals were removed from the results to limit the documents to journal articles, totaling 500. The journal articles were further limited to articles (439), reviews (28), and editorials (14). Thereafter, the articles were “eyeballed” by scanning through the titles and abstracts to remove irrelevant articles. The final list contained 153 articles. The VOSViewer was used to analyze the networks and clusters of urban computing research after noting their influential contributions and trends (Figure 1).

The second part of the review involved limiting the search to articles on Saudi Arabia, which returned 29 articles. These articles, government publications, and news articles were reviewed to analyze and discuss urban computing in the context of Saudi Arabia. The analysis was done by using the urban computing framework extracted from the literature.

## 3 Results and discussion

### 3.1 The trend and influential contributions to urban computing

**Figure 2:**
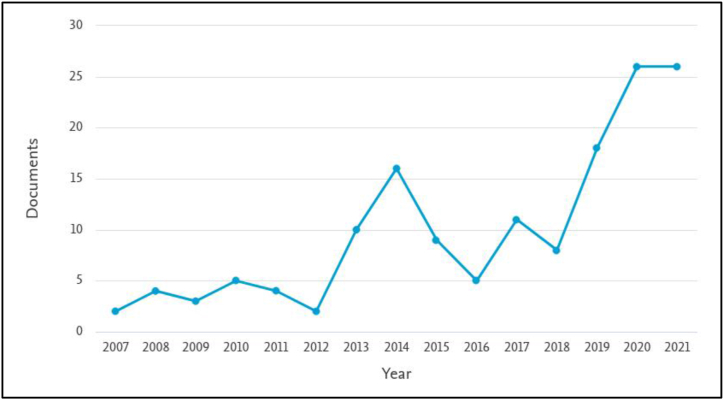
The trend of urban computing article publication.

**Figure 3:**
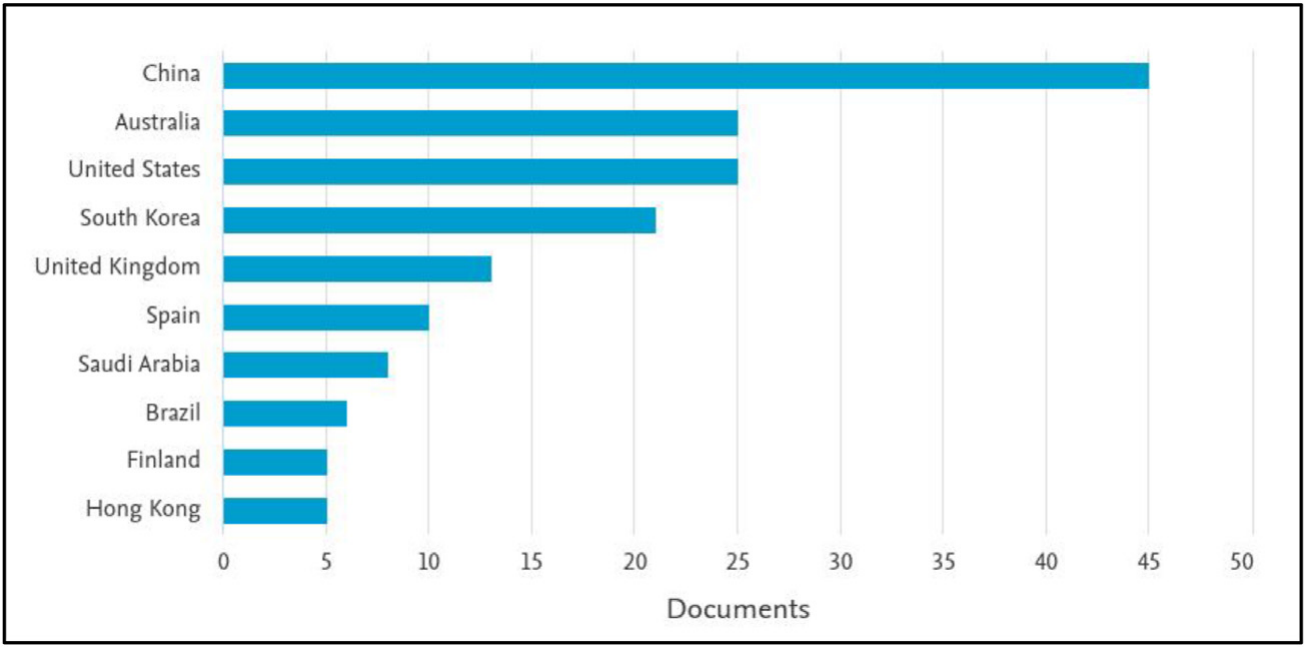
The top ten countries to contribute to urban computing research.

**Figure 4:**
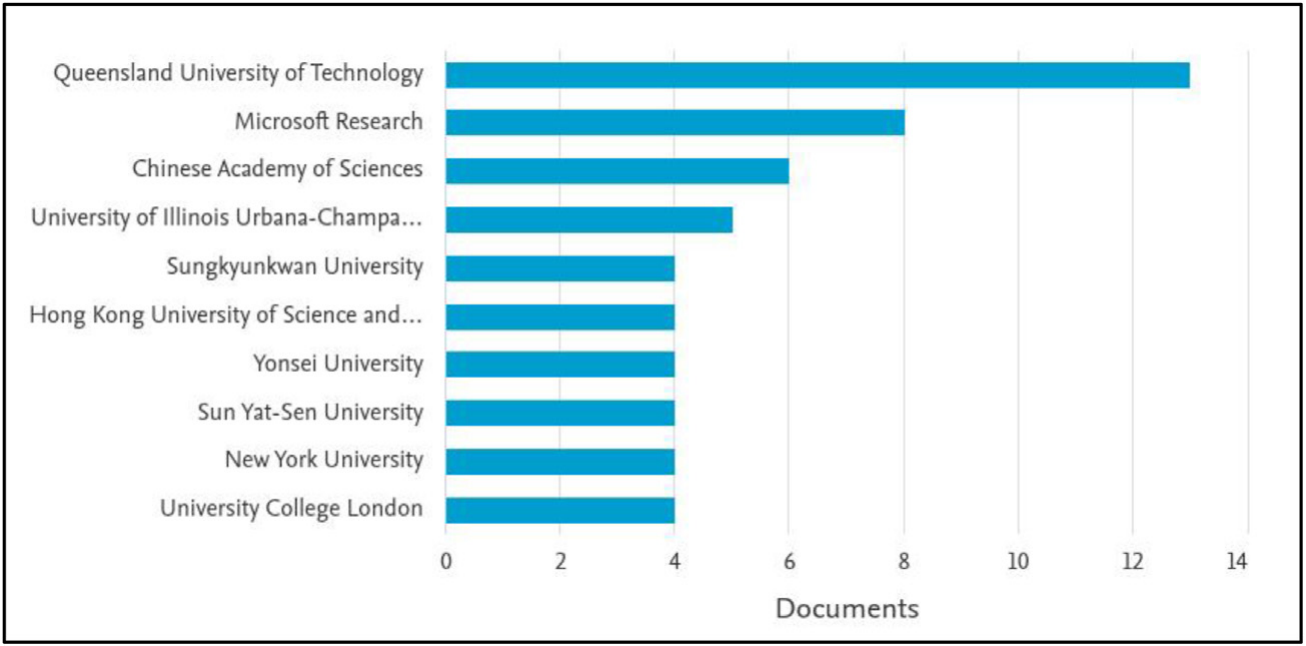
The top ten institutions to contribute to urban computing research.

**Figure 5:**
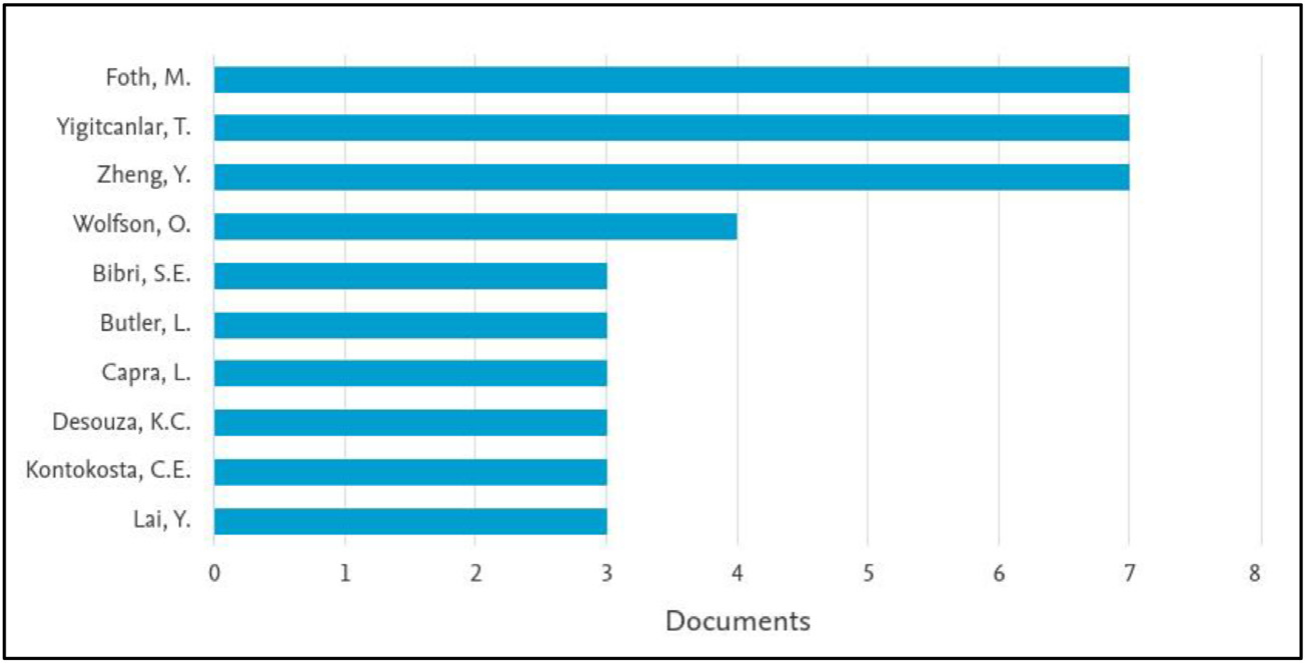
The top ten authors to contribute to urban computing research.

The number of articles published on urban computing indicates an increasing interest in the subject, from two articles in 2007 to 26 articles in 2021 (Figure 2). China has made the largest contribution to this list (45) with Saudi Arabia on number seven (7) (Figure 3). Figure 4 indicates that the Queensland University of Technology in Australia made the highest contribution (13) with Microsoft Research at second (8). This shows the private sector's interest in urban computing research. Figure 5 highlights the contribution of authors, with Marcus Foth, Tan Yigitcanlar, and Yu Zheng being the leading contributors.

**Figure 6:**
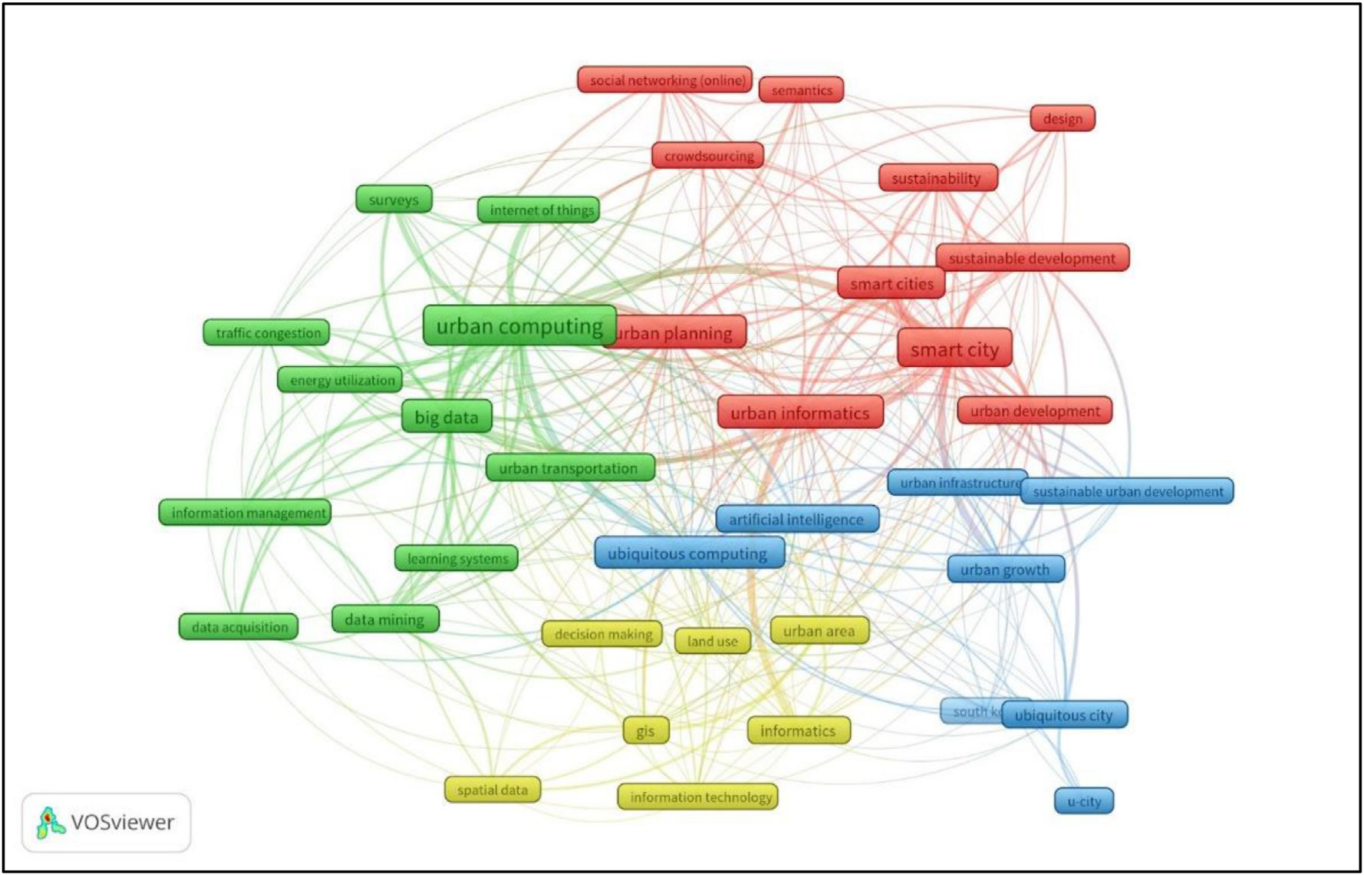
Clusters of urban computing research.

The result of the VOSviewer co-occurrence analysis showed four clusters of urban computing research based on the highest occurring keywords in the clusters – urban computing (11 keywords), smart city (11 keywords), ubiquitous city (8 keywords), and urban area (7 keywords) (Figure 6). The keywords under each cluster indicated the trend of urban computing research. Big data, traffic congestion, IoT, and energy utilization were part of the urban computing keywords. Smart city keywords included sustainability, urban informatics, crowdsourcing, social networking, and urban planning. Land use, Geographic Information System (GIS), spatial data, and information technology (IT) appeared in the urban area keywords. Ubiquitous city keywords included artificial intelligence (AI), ubiquitous computing, urban growth, urban infrastructure, and sustainable urban development (Table 1).

**Table 1:** Citation counts and issues addressed by urban computing clusters.

Cluster	Keyword (occurrence)	Topics addressed, references, and citation counts	
Urban computing	Urban computing (52), Big data (26), IoT (7)		Trajectory data mining (Zheng, 2015; Shang et al., 2014)∗ (1071; 127), Urban computing (Zheng et al., 2014a, 2014b; Kindberg et al., 2007)∗ (648; 280; 102), Taxi sharing system (Santi et al., 2014; Ma et al., 2014)∗ (410; 231), Smartphones as sensors for monitoring and data mining (Su et al., 2014) (274)
Smart city	Smart city (37), Urban informatics (26), Urban planning (21)		Smart cities (Ismagilova et al., 2019; Yigitcanlar et al., 2019)∗ (304; 255), Smart city services (Lee and Lee, 2014)∗ (171), Ethics of smart city (Kitchin, 2016)∗ (168), Crowdsourcing smart ideas (Schuurman et al., 2012)∗ (163), Smart city transformation (Kumar et al., 2020)∗ (121), Urban informatics (Lu et al., 2020)∗ (109), Crowdsourcing big data for emergency events (Xu et al., 2020)∗ (93)
Ubiquitous city	Ubiquitous city (11), Ubiquitous computing (17), Urban infrastructure (5)		Ubiquitous city (119) (Shin, 2009), Artificial intelligence (98) (Yigitcanlar et al., 2020), Data sharing in ubiquitous cities (96) (Shen et al., 2017)
Urban area	Urban area (10), GIS (9), Information technology (7)		Sensing urban land use with Twitter (142) (Frias-Martinez and Frias-Martinez, 2014),

∗ Respective citations of the articles.

The analysis of the citations with 100 or more counts indicated that urban computing articles had the highest citations (410–1071 citations) (Table 1). They addressed issues such as the concepts, definition, and applications of urban computing (Zheng et al., 2014a, 2014b; Kindberg et al., 2007), trajectory data mining (Zheng, 2015; Shang et al., 2014), and vehicle pooling (Santi et al., 2014; Ma et al., 2014). However, the smart city cluster was the most active and updated. The research articles in this cluster are relatively recent with high citations and include topics like smart city transformation (Kumar et al., 2020), urban informatics (Lu et al., 2020), and big data for emergency events (Xu et al., 2020).

### 3.2 Urban computing: achieving widespread smart city development

As mentioned previously, smart technologies and the IoTs are tools to reach sustainability and progressively lead our cities to resilience and energy conservation. Furthermore, it is essential to know our cities and their unique challenges to implement the right smart strategies and methods to obtain sustainability. However, it is not about the smart technologies alone, it is about collecting the relevant data to reach the solutions and managing that data and connecting technology to resolve city challenges. Another relevant aspect is determining the right places for data collection since large-scale data collection may be a challenge due to limited resources.

All these concerns can be addressed with urban computing and it is indeed the way forward for Saudi cities to become smart cities. Torres-Ruiz and Lytras (2016: pp. 113) defined urban computing as “the technology for acquisition, integration, and analysis of big and heterogeneous data generated by a diversity of sources in urban spaces, such as sensors, devices, vehicles, buildings, and human, for tackling the major issues that cities face.” The rapid advancement of urbanization has changed people's lives for the better, however, it has also resulted in major concerns such as high energy consumption, traffic congestion, and pollution. The goal of urban computing is to address these complexities and difficulties by using technologies and data accumulated from the cities (Javidroozi et al., 2019). Bibri (2021: pp. 4), while examining the data-driven nature of urban computing for solving urban sustainability problems, defined urban computing as “using a set of sensors, devices, systems, platforms, infrastructures, networks, and the associated algorithms, techniques, processes, and protocols for the purpose of addressing and overcoming the issues engendered by the negative consequences of urbanization and the complex challenges of sustainability”.

Urban computing focuses on lifestyles and technologies adopted within the context of urban public spaces. We are at present living in the technologically-enhanced generation of social media where user-generated content interaction and communication focus on increasing the sustainability of smart cities (Torres-Ruiz and Lytras, 2016). Since urban computing eliminates inefficiencies and improves the ability to respond to the needs of the people through the smart city construct, it can help Saudi cities to overcome varied urban difficulties and achieve sustainability.

To enable service improvement and expansion, urban infrastructures are becoming more reliant on networked technology. The transformative digitalization can, consequently, result in large volumes of data “digital footprints”, which can be utilized to improve the knowledge of human behavior and enhance the livability of urban areas (Oliver, 2011; Zhu et al., 2022). Furthermore, the digital transformation of cities through urban computing is an interdisciplinary field where computer science intersects with varied city-related disciplines, including civil engineering, transportation, economics, energy engineering, environmental sciences, ecology, and sociology. This study focuses on three urban issues, derived from the literature, that are obstacles to sustainability in Saudi cities – traffic congestion or mobility, poor air quality, and high energy consumption (Aina, 2017; Abubakar and Aina, 2018; Abubakar and Dano, 2020).

**Figure 7:**
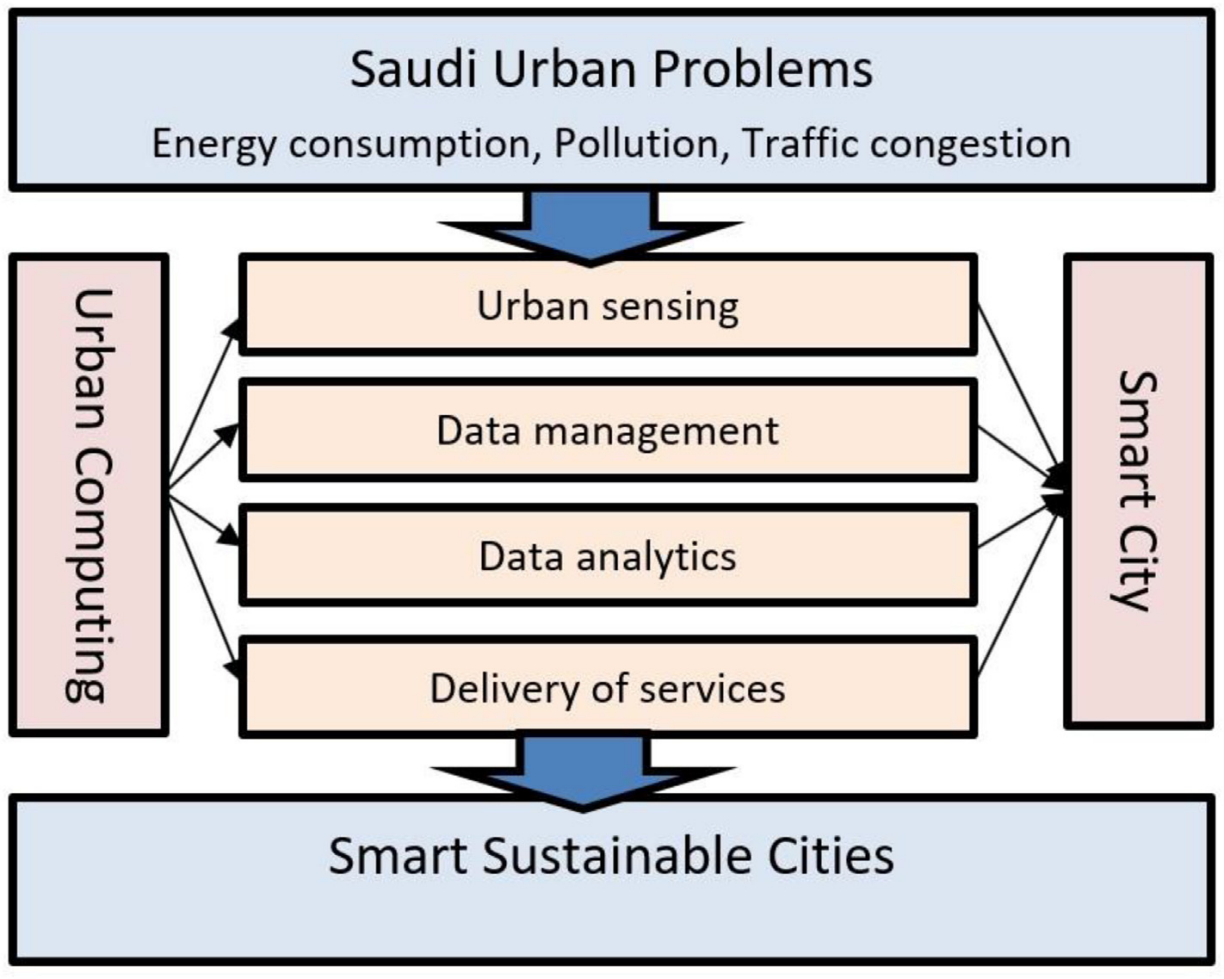
A conceptual framework for applying urban computing.

Urban computing has four main characteristics – urban sensing, data management, data analytics, and delivery of services (Kontokosta, 2021; Wu et al., 2022; Yang et al., 2022; Zheng et al., 2014a; Zhang et al., 2022). This study will explore these characteristics and examine the transformation of Saudi cities into smart, sustainable urban areas by analyzing each component and deducing the possible implications for Saudi (Figure 7).

### 3.3 Using the urban computing framework to examine Saudi smart city development

The policy direction for smart city development in Saudi Arabia is derived from Saudi Vision 2030 (www.vision2030.gov.sa). One of the strategic objectives of Vision 2030 is to have a vibrant society through the improvement of livability in Saudi cities and the promotion of environmental sustainability. The government is acting on this policy objective by promoting smart city initiatives. The Ministry of Municipal and Rural Affairs and Housing is targeting the transformation of selected Saudi urban areas to smart cities through public and private partnership (Saudi Gazette, 2015). Smart city development is still evolving in Saudi Arabia compared to smart cities in the USA, Europe, Japan and Singapore, which have been ranked as topmost smart urban areas (Ozkaya and Erdin, 2020). There has been an increasing investment in digital infrastructure and ongoing plan to deploy autonomous vehicles (Saudi Gazette, 2021a, Saudi Gazette, 2021b). The recent plan to build a multi-billion-dollar smart sustainable city (NEOM) might further boost the development of smart cities in other urban areas which have been facing challenges including funding (Aina et al., 2017). Urban computing could help the cities in addressing the challenges through incremental solution-driven smart development. The areas of urban computing research in Saudi Arabia are mainly health applications including COVID-19 pandemic (Al-Jehani et al., 2021; Alomari et al., 2021a; Alotaibi et al., 2020), big data and smart mobility (Alomari et al., 2021b; Alharbi et al., 2021; Al-Nabhan et al., 2021; Aljoufie and Tiwari, 2022), cybersecurity and IoT (Alhalafi and Veeraraghavan, 2021; Alanazi and Soh, 2020; Choo et al., 2021), and NEOM (Nuseir et al., 2020; Alotaibi et al., 2019).

#### 3.3.1 Urban sensing

Since the emergence of the IoT and advancements in sensing technologies, cities have been generating a huge amount of data for the twin purpose of building the environment and offering richer services to the citizens (Kontokosta, 2021). In addition, cities have several factors that need measuring and sensing, like air pollution, weather, traffic patterns, and road quality. Thus, urban sensing technologies play a significant role in developing advanced analytic solutions for urban managers and decision-makers.

This section examines how an area of interest, such as traffic patterns, can be measured by attaching sensors at various locations and on objects to solve urban issues. The primary function of these sensors is to collect several types of data, such as examining people’s mobility and routing behavior in the road network by using GPS-based sensors or mobile phone signals. Hyper-local measurements can be obtained for huge chunks of a city by adding affordable sensors to crowdsourced urban cars. These and similar strategies can support Saudi cities in mitigating urban issues such as mobility and traffic congestion.

New York City developed a data sensing strategy and attached sensors to taxis. They demonstrated that taxi fleets have a surprising amount of sensing power. In a single day, ten taxis covered one-third of Manhattan’s streets. In addition, taxis' detecting power is consistent among cities across the world (O'Keeffe et al., 2019). This model can help city planners and policymakers quantify the required number of mobile sensors to cover different urban areas and temporal coverage requirements. Moreover, sensors on automobiles equip cities with low-cost, effective sensing capabilities.

The sensors can be more effective if they are networked and have the computational capacity to provide meaningful information from the data collected, thereby, fostering environmental sustainability (Andronie et al., 2021; Cooper et al., 2021; Mulligan, 2021). In addition, autonomous vehicle perception sensor data can be utilized to make networks of connected vehicles for traffic analytics and management to improve smart mobility and sustainability (Woodward and Kliestik, 2021; Aldridge and Stebel, 2021). Mobility improvement can lead to competitiveness and economic gains (Poliak et al., 2021a,b).

It is only when we study the risks and challenges of urban sensing development that we can achieve a successful outcome. The primary challenge is loosely controlled and non-homogeneous distributed sensors. A traditional, non-networked sensor can be configured to send readings at a certain frequency. However, the amount of data collected cannot be controlled since the people providing the data are uncontrollable. Therefore, there may be a lack of data sharing. Moreover, a lack of people during certain periods inevitably results in missing data and sparsity problems. On the contrary, when there is a large amount of data from user-generated content for one location, there could be an overwhelming amount of data or even redundant data, which would add to the sensing, communication, and storage workload.

Thus, it can be concluded that data sensing purely relies on the people and the location. Only when a focused area of interest is decided, the relevant methodologies and strategies to understand and resolve possible risks can be executed.

#### 3.3.2 Data management

Smart city data comes from varied sources. They contain sensitive information and are marked by quality difficulties. Social media and human mobility data are sources of data management, which are organized by an indexing structure that integrates spatio-temporal and textual information for efficient data analysis (Rathore et al., 2018; Liu et al., 2021).

Since data sensing may have security concerns, data management can be categorized based on data sensitivity. Data can be managed more efficiently and effectively if categorized properly. This can be done by creating a framework that seeks to be a generic solution for data fusion and management in smart cities. To safeguard data privacy, the proposed technique divides the data into three categories – sensitive, quasi-sensitive, and open data (Liu et al., 2017). Sensitive data can be used to directly identify an individual, such as a social security number. Quasi-sensitive data can be used to identify an individual when linked to other sources like social media, which include age, gender, address, etc. Open data is freely available to the public and can be accessed quickly. Different strategies are used to govern the sharing and dissemination of data based on their sensitivity and classification.

#### 3.3.3 Data analytics

Certain components in a smart city need critical attention, such as integrating all smart systems (smart homes, smart parking, etc.) and IoT devices (sensors, actuators, and smartphones). However, when a large number of IoT gadgets are connected to collect urban data over the internet, a large amount of data is generated. Thus, when a large volume of high-speed data is collected to be processed and analyzed through numerous sources (e.g., real-time sensors, remote sensing, online streaming, social web networks, e-health, etc.) with varied structures and types, it is referred to as big data (e.g., IoT, Machine-to-Machine, Databases, Internet-based voice and media calls, Wireless Sensor Networks, etc.) (Rathore et al., 2018).

Big data is defined by three fundamental characteristics – big volume, high velocity, and a vast variety (Furht and Villanustre, 2016; Hassan et al., 2021). In addition, it is “exhaustive in scope, scalable, flexible, relational in nature, indexical in identification, and has a high resolution” (Kitchin, 2014: pp. 2). These features assist in city data analysis and the results can be used effectively for smart transformation and city management.

If, and when, an anomaly occurs, data analytics can be used to find the accurate places where mobility patterns diverge significantly from the original. In addition, the anomaly can be characterized by extracting representative phrases, connected to regions and periods, from social media (Zhang et al., 2020).

This stage is crucial since it needs an extensive amount of work and focus to guide the data toward maximum benefit. Saudi cities need data analytic investments and stockholders, such as telecommunication authorities and decision-makers, to support this critical stage.

#### 3.3.4 Delivery of services

The final step of the urban computing process is the delivery of services where all the data gathered in the previous steps is investigated to build smart city services and solutions. Data is integrated into higher-level services or apps during this process, which improves the quality and performance of smart city services. The position and description of the anomaly are provided to the surrounding cars so that they can bypass it on the road. In addition, the data is sent to the transportation authority who can use it to disperse traffic and diagnose the problem (Zheng et al., 2014a). Recent research has examined the ways to make AI devices more human-like in delivering services (Pelau et al., 2021). The aim is that the devices would provide useful computation results and emphatically deliver services.

By addressing urban concerns, such as traffic congestion, pollution, disaster mitigation, aging population, big infrastructure maintenance, and housing, urban governance strives to improve the effectiveness and efficiency of urban management and decision-making. The goal is to provide the inhabitants with a better day-to-day lifestyle. Urban computing assists people to interpret data and extract actionable knowledge and other analytical results to alleviate urban difficulties and provide services to meet urban governance and urban services aims (Shi and Zhang, 2021).

There are multiple ways to use the collected data for the city’s benefit. Data collected from traffic lights can help traffic flow management in a certain area or data collected on air quality performance from certain locations can help find the cause and the solution. This can help Saudi cities enhance service quality. It requires the collaboration of several entities and authorities to build a sustainable service.

#### 3.3.5 Urban computing in the context of Saudi Arabia

Urban computing in Saudi Arabia is a new field, which opposes several challenges of a smooth transition to a smart city. Urban sensing is the use of technology, such as sensors, to collect data throughout the city. There is a huge increase in the amount of available data from varied sources – real-time, satellite images, signals, transit, and social media. The challenge is to find ways to apply computational techniques to the data coming from the cities and convert them into information for urban purposes. A smart city has a large number of smart sensors that generate a large amount of data for varied uses. However, collecting data from these sensors presents several obstacles, including connecting the sensors to the data center via a communication network, which requires expensive infrastructure. Nevertheless, due to its advantages, such as data offloading, operations, and communication on asymmetric links, simple strategies such as connecting smart sensors with data centers on a broad scale via smart vehicles (transport fleets or taxi cabs) is a good place to start for an efficient and economical collection of everyday data (Naseer et al., 2021).

Data management is crucial when implementing urban computing in the Saudi context. It can help analyze data faster and more efficiently. The urban data management layer uses cloud computing platforms, indexing structures, and retrieval algorithms to manage large-scale and dynamic urban data from various domains, such as traffic, meteorology, human movement, and points of interest (PoI) (Zheng, 2019). This layer devises cloud storage strategies that Saudi Arabia must consider. However, the security and privacy of the collected data are crucial at this stage. It is important to provide privacy and security features for smart city applications in Saudi. Furthermore, since urban computing would transition Saudi cities to smart cities, citizens’ involvement and trust are important. If the citizens are unwilling to contribute and engage in the creation of smart cities, the notion will remain incomplete. Therefore, user privacy and data security must be maintained at all times. It must be noted that security is not absolute but dynamic; the fundamental and phenomenal strategy for preventing attacks on smart cities and their people must evolve.

As mentioned previously, data analysis needs extensive work. The Saudi Data and AI Authority (SADIA) is a great example of analyzing and using the collected data to provide services to the Saudi people. They have multiple products that advance data analytics in Saudi Arabia, such as the Estishraf Platform. By investing in huge data analysis capabilities and AI, through a national multi-specialization team, the Estishraf Platform provides future visions and projections to decision-makers in the kingdom. This could lead to the development of a thorough understanding of economic, strategic, and social aspects to make decisions and evaluate the performance indicators of various entities in support of Vision 2030. Data analytics demands advanced technologies to integrate real-life information into computerized data to understand certain patterns and unrevealed information.

Urban computing provides major benefits during the delivery of services. It takes Saudi cities’ urban issues and provides a set of services to improve the quality of life through the data collected and analyzed in the previous three stages. One of the applications of urban computing that could benefit Saudi cities is the transport network system. The system challenges can be addressed by collecting, managing, and analyzing all the required data to provide a specific service, such as creating new traffic routes to avoid congestion. A simple strategy that can be applied to Saudi is the installation of sensors in personal vehicles, taxis, and buses to collect and analyze traffic data. They provide updates to commuters via cell phones after analyzing the data to reduce the growing traffic and congestion. Hence, IoTs reduce the cost of road development. The service-providing layer handles the challenge of connecting urban computing with Saudi city-related topics, such as urban planning, environmental theory, and transportation. Therefore, a collaborative set of a sector is needed to move towards the betterment of Saudi cities.

### 3.4 Urban data

This section presents data on urban computing to evaluate the knowledge that may be obtained in smart cities through the integration of independent data sources. The sources of data could be land use, waterways, water barriers, buildings, roads, amenities, PoI, weather, traffic, pollution, or parking lot data. However, the sources discussed in this paper are geographical, environmental monitoring, and energy. In addition, the challenges of using these data sources are mentioned briefly.

#### 3.4.1 Geographical data

Geographical data is needed to assess city components or plans to resolve urban issues. It may contain multiple sources, such as road network data, PoI data, and land use data.

A road network data set is information about a road network, which is a system of interconnected roads designed to accommodate automobiles and pedestrians. This data is the most frequently-used geographical data in urban computing. It includes data for traffic monitoring and predictions, routing, urban planning, and energy consumption analysis. These data can be represented through a graph that comprises a set of edges, which denotes road segments, or nodes for road intersections where each node has a unique identity and geospatial coordinate. This representation helps analyze and know the data collection points for optimum utilization and efficient outcomes. The development of an intelligent transport system for university campus commutes is an example of such an outcome (Qoradi et al., 2021).

#### 3.4.2 Environmental monitoring data

Cities face several problems that imply massive challenges. For instance, many developing countries are fighting air pollution due to an increase in automobiles and urbanization. Governments have built air quality monitoring stations in the cities to inform the people about the concentration of air pollutants. Saudi Arabia’s cities have been facing air pollution problems. The environmental monitoring data is an important application to mitigate the consequences of air pollution in Saudi cities. Environmental data must be collected, such as meteorological data (humidity, temperature, barometer pressure, wind speed, and weather conditions), which can be found on public websites. Air quality data, such as the concentration of PM_2.5_, NO_2_, and SO_2_, can be obtained from air quality monitoring stations (Zheng, 2019). Gases such as CO_2_ and CO can be detected through portable sensors that absorb enough air to provide insight into the air quality.

Zheng et al. (2014a) highlighted how air quality information can be analyzed and presented at different scales. Data can be collected by building ground-based air quality measurement stations. A measurement station location may have limited nodes, however, it will still be helpful to monitor the air contaminant levels. In addition, real-time and fine-grained air quality information throughout a city can be depicted (Zheng et al., 2014a). Furthermore, existing monitor stations and a range of data sources, such as meteorological, traffic flow, human mobility, road network structure, and POIs, also report air quality data. Fine-grained air quality data could assist people to decide appropriate jogging spots, the right time to close a window or the time and place to wear a facemask. This is important for people's health and overall well-being since it gives them a perspective on locations that need attention. In addition, such data can be utilized to propose additional monitoring station sites if the current ones are insufficient.

Another environmental data that is helpful to citizens is noise data. Since cities are the major contributors to noise pollution and as traffic and other noise-generating activities increase in the Kingdom of Saudi Arabia (KSA), negative health and other consequences are expected. A study was conducted in a region in Jeddah about noise pollution. The study aimed to see if the data available in the KSA municipalities was adequate for developing urban noise maps and if the present environmental noise mapping and noise annoyance models applied to the KSA. As a result, using commercially-available noise mapping software, noise maps were created for the Al-Fayha District in Jeddah City, Saudi Arabia (Zytoon, 2016).

The analysis of the study is that most of the data needed for traffic noise forecast and annoyance analysis were available either through the municipality's GIS department or through other government agencies. The predicted noise levels were found to be greater, during the afternoon, evening, and night, than the maximum recommended values stated in the KSA environmental noise rules. According to the annoyance analysis, depending on the planning zone and period of interest, substantial percentages of district residents were severely upset. As a result, there is an urgent need to consider environmental noise reduction in the KSA national plans, which can be supported and achieved through urban computing strategies.

#### 3.4.3 Energy data

The increased amount of energy consumption due to rapid urbanization needs immediate attention. Thus, technologies that can detect city-scale energy costs and contribute to the improvement of energy infrastructures are needed to minimize energy consumption.

Automobiles are major energy consumers. The amount of gas consumed by vehicles on roads and at gas stations reflects a city’s overall energy consumption and greenhouse gas emission (Rahman et al., 2017). The corresponding data can be obtained directly through sensors, such as those used by insurance companies to collect data from a vehicle. In addition, data can be deduced from other sources, such as the GPS trajectories of a vehicle. The information can be used to assess a city’s energy infrastructure, such as gas station distribution, estimate pollutant emissions from automobiles on roads, and determine the most efficient problem-solving path (Goel et al., 2015).

Buildings, such as apartment and office buildings, too need energy monitoring. A strategy such as smart grid technology can be used to monitor energy consumption, using electronic controlling, metering, and monitoring in electricity infrastructure (Zafar et al., 2020). Furthermore, smart meters and sensors have been implemented in several electrical grids in recent years for generating electricity consumption, transmission, and distribution data. The electricity consumption of an apartment or a building can be utilized to optimize domestic energy consumption by shifting peak loads to low-demand hours through an energy management system (Llaria et al., 2021). These methods can help assess reasonable energy consumption levels of different sectors and mitigate several urban issues currently facing Saudi cities.

Saudi Arabia has achieved remarkable milestones in using urban computing to enhance smart city transformation. The major strides are in service provision. Reportedly, more than 875 government services have been digitalized (Aina et al., 2019a). One of the e-services applications, Tawakkalna, was awarded the United Nations Public Service Award 2022 for its usefulness during the COVID-19 pandemic (Arab News, 2022). As mentioned above, sensors have been deployed in big cities to improve air quality monitoring. For example, a network of about 32 stations was developed in Riyadh (Aina et al., 2019a). However, the implementation is yet to match the level achieved by some other countries, whereby, the data and air quality status are available online for monitoring and research.

## 4 Conclusion

Cities nowadays are going through rapid urbanization that has forced the people and the authorities to rethink their perception of cities, especially Saudi cities. Currently, urban areas in Saudi Arabia are facing environmental and urban issues due to population increase, such as traffic congestion, poor air quality, water and air pollution, and climate change.

Possible mitigation of these crises can occur through a smart and sustainable city concept. The IoT and technologies must be used as a tool to increase sustainability and a better quality of life. Urban computing can help us plan for smart and sustainable cities. Urban computing is comprised of four phases – urban sensing, data management, data analytics, and delivery of service. These characteristics were derived from multiple books and journal articles to simplify the broad idea of urban computing.

Each characteristic has its strategies that create a unique path to achieving smart and sustainable Saudi cities. Starting with urban sensing, which is the use of technologies to gather and collect data from the urban context, this stage has its challenges, such as missing data, redundant data, and excessive data. However, if the challenges are resolved, valuable data can be gathered to achieve sustainability. During data management, which refers to the efficient structuring of data for proper analysis, protection of data and privacy are major challenges. Categorization of data as sensitive, quasi-sensitive, and open can help protect and secure data. Data analytics, which is the most crucial stage of urban computing, requires Saudi authorities’ commitment and technology investments such as in big data analytics systems. This saves time and effort to analyze urban data and helps understand unrevealed patterns. Delivery of service focuses on the comprehensive view of all the stages of urban computing and deducing solutions and services for urban issues. This stage addresses citizens’ concerns and focuses on their needs. Saudi cities need to focus on citizen involvement for transformation to smart cities to achieve fast and efficient sustainability.

Urban data that have helped to foster smart sustainable cities, by providing more insights into urban complexity, include environmental data monitoring, energy data, and geographical data. Geographical data contains several sources, such as road network data, PoI data, and land use data. Environmental data monitoring requires the building of air quality monitoring stations in cities to inform people of the air pollutant concentration. Energy data can detect city-scale energy costs and monitor energy consumption to improve energy infrastructures.

All these examples can be used in the context of urban computing to help achieve smarter and more sustainable cities. Saudi cities have the potential to transform into smart cities using urban computing, evident from its achievements in service provision and environmental monitoring. When constructing the necessary infrastructure for the implementation of urban computing, it is important to identify all the challenges and possible risks, study and develop an implementation plan, and develop a maintenance plan.

## Declarations

### Author contribution statement

All authors listed have significantly contributed to the development and the writing of this article.

### Funding statement

The APC fund is provided by the King Fahd University of Petroleum and Minerals, Saudi Arabia.

### Data availability statement

Data will be made available on request.

### Declaration of interest’s statement

The authors declare no conflict of interest.

### Additional information

No additional information is available for this paper.
